# Adverse effects of psychedelics: From anecdotes and misinformation to systematic science

**DOI:** 10.1177/02698811211069100

**Published:** 2022-02-02

**Authors:** Anne K Schlag, Jacob Aday, Iram Salam, Jo C Neill, David J Nutt

**Affiliations:** 1 Drug Science, London, UK; 2 Department of Brain Sciences, Imperial College London, London, UK; 3 Department of Geography, King’s College London, London, UK; 4 Department of Psychology, Central Michigan University, Mount Pleasant, MI, USA; 5 Department of Psychiatry and Behavioral Sciences, University of California, Davis, Sacramento, CA, USA; 6 Division of Pharmacy and Optometry, School of Health Sciences, The University of Manchester, Manchester, UK

**Keywords:** Psilocybin, dimethyltryptamine, ayahuasca, mescaline, d-lysergic acid diethylamide, abuse liability/dependence, hallucinogen persistent perception disorder, toxicity, hypertension

## Abstract

**Background:**

Despite an increasing body of research highlighting their efficacy to treat a broad range of medical conditions, psychedelic drugs remain a controversial issue among the public and politicians, tainted by previous stigmatisation and perceptions of risk and danger.

**Objective:**

This narrative review examines the evidence for potential harms of the classic psychedelics by separating anecdotes and misinformation from systematic research.

**Methods:**

Taking a high-level perspective, we address both psychological and psychiatric risks, such as abuse liability and potential for dependence, as well as medical harms, including toxicity and overdose. We explore the evidence base for these adverse effects to elucidate which of these harms are based largely on anecdotes versus those that stand up to current scientific scrutiny.

**Results:**

Our review shows that medical risks are often minimal, and that many – albeit not all – of the persistent negative perceptions of psychological risks are unsupported by the currently available scientific evidence, with the majority of reported adverse effects not being observed in a regulated and/or medical context.

**Conclusions:**

This highlights the importance for clinicians and therapists to keep to the highest safety and ethical standards. It is imperative not to be overzealous and to ensure balanced media reporting to avoid future controversies, so that much needed research can continue.

## Introduction

Psychedelic medicines are a rapidly developing area of clinical research (Nutt and Carhart-Harris, 2021) and public health policy. Clinical developments, together with changes in public interest, are increasingly leading to substantive changes at the regulatory level in the United States and Canada (Aday et al., 2020a). Within the past 3 years, psilocybin and other organic psychedelics have been decriminalised in Denver, Colorado; Oakland, California; Santa Fe, California; Ann Arbor, Michigan; Somerville, Massachusetts; Washington, D.C.; and the state of Oregon. Going beyond decriminalisation, Oregon voters recently passed a bill giving the Oregon Health Authority 2 years to develop a division to regulate the production, distribution, administration and possession of psilocybin.

In Canada, last year, the Minister of Health gave approval on a case-by-case basis for several terminally ill patients to receive psilocybin for the purposes of treating end-of-life distress (Lozano, 2020). Successful preliminary results led Health Canada to announce in December 2020 their intention to expand the Special Access Programme (SAP), so that practitioners could, on behalf of patients with serious or life-threatening conditions, request access to restricted drugs. This change would significantly broaden the number of individuals permitted to access psychedelic therapy. Subsequently, Health Canada granted exemption to 16 healthcare professionals to take psilocybin themselves for personal training (Dubinski, 2020), which is indicative of a rapidly growing infrastructure for psilocybin-assisted therapy in Canada. In Europe, a special use programme for d-lysergic acid diethylamide (LSD) and psilocybin has been established in Switzerland to provide compassionate access to (mainly major depression and post-traumatic stress disorder (PTSD)) patients not responding to other treatments (Schmid et al., 2021).

Although these changes in regulation suggest that the stigma surrounding psychedelics may be dissipating, still many misconceptions exist. This narrative review aims to separate the anecdotes and misinformation from the systematic evidence. We cover the classic serotonergic psychedelics (5-HT2A receptor agonists, referred to ‘psychedelics’ henceforth): these are plant-derived medicines; psilocybin, N, N-dimethyltryptamine (DMT), ayahuasca, mescaline and those synthesised in the laboratory, LSD. We exclude n-BOMEs, 3,4-methylenedioxymethamphetamine (MDMA), ketamine and ibogaine, as these are distinguished from classic psychedelics, both in their effects and in their pharmacology. We focus on the effects of full psychedelic doses; for an in-depth review on microdosing psychedelics, please see Kuypers et al. (2019). For more details on the pharmacology and neuroscience of the drugs we discuss, please see Nutt et al. (2020).

Assessing the risks of psychedelic use is challenging, as there are many different substances, applications, environments and population groups in this rapidly developing field. This article looks at the potential adverse effects of psychedelics, using the current science to outline risks as well as anecdotes surrounding harms. Many of these risk perceptions originate from the first wave of psychedelic repression in the middle of last century often with sensationalised media reports. Yet, these still contribute to their current stigmatisation.

Johansen and Krebs (2015) propose that modern anti-psychedelic legislation began over 100 years ago when rival religious groups campaigned against Native American peyote use, calling peyote ‘addictive’ as well as an ‘insidious evil’ (Newberne and Burke, 1922). Although evidence and human rights arguments led to exemptions for specific indigenous groups, the laws and biases against peyote remained in place and were then extended to other psychedelics.

Still today, psychedelics attract emotive and often polarised opinions (Rucker et al., 2018). It is essential to address this issue now as psychedelics are increasingly shown to treat a broad range of hard to treat disorders, with the potential to treat many more.

For our review, we gave precedence to randomised controlled trials (RCTs), systematic observational data collections and systematic reviews. Except where compelling, we avoided individual case reports for reasons outlined in detail by Krebs and Johansen (2013), such as the frequent failure to rule out pre-existing conditions or the use of other drugs, which could have contributed to adverse effects after psychedelic use.

### LSD

First synthesised by Albert Hofmann in 1938, LSD is a semi-synthetic tryptamine derived from the naturally occurring ergot alkaloid ergotamine (Nichols, 2004). It acts primarily as a serotonergic receptor agonist and also acts at dopaminergic and adrenergic receptor sites (Nichols, 2004). Having been described as a ‘problem child’ (Hofmann, 1979), LSD became a major focus of negative public perceptions, many of which still prevail today.

By 1961, a large body of research with LSD in humans, incorporating over 1000 papers, including over 40,000 participants, had accumulated (Nutt et al., 2013). Although studies were small, they reported largely positive effects and a lack of adverse effects (as reported by the clinician). However, significant shortcomings were outlined in a review at that time (Savage et al., 1966), including lack of appropriate controls, small numbers of participants, inappropriate statistical analyses and importantly lack of follow-up, which has been rectified in recent trials. Unfortunately, for this promising field of research, however, the 1960s experimentation with LSD (and to a lesser extent, psilocybin), by infamous Harvard psychologists Richard Alpert and Timothy Leary, and the emergence of 1960s counterculture led to a media frenzy and sensationalised representations of these substances, contributing to the halt of promising scientific research and national and international (under the 1971 UN Conventions) bans on LSD. Once LSD was banned, most countries made other serotonergic psychedelics illegal as well (Nutt et al., 2013; Rucker et al., 2018).

### Psilocybin

Psilocybin is found in over 200 species of mushrooms. Psilocybin-containing mushrooms have been used for religious purposes throughout Mesoamerica for centuries (McKenna and Riba, 2016), with mushroom-shaped artefacts dating back to at least 500 BC (Guerra-Doce, 2015). In the West, psilocybin was first isolated in 1958 (Hofmann et al., 1958). While it was the lesser studied psychedelic in the 1960s in comparison to LSD, it has been the focus of much contemporary research, including as an efficacious treatment (combined with psychotherapy) for obsessive compulsive disorder (OCD) (Moreno et al., 2006), treatment-resistant depression (TRD) (Carhart-Harris et al., 2016), smoking cessation (Johnson et al., 2014) and alcoholism (Bogenschutz et al., 2015), leading to an increasing body of positive evidence being developed. The first RCT comparing psilocybin to a conventional selective serotonin reuptake inhibitor (SSRI) antidepressant found the former to be as efficient at reducing symptoms of depression, and with fewer side effects (Carhart-Harris et al., 2021). However, sample sizes remain small, and further research – using rigorous methodologies to address issues, such as blinding – is required to further understand the (long-term) effectiveness of these treatments.

### Mescaline

Mescaline (3,4,5-trimethoxyphenethylamine) was first isolated from *Lophophora williamsii*, the peyote cactus, in 1896 by Arthur Heffter, making it the first naturally occurring psychedelic alkaloid to be isolated in the laboratory (Heffter, 1998). Peyote has a long history of religious use, dating back at least 5700 years (El-Seedi et al., 2005). Compared to the other classic psychedelics, contemporary research with mescaline has remained relatively limited to date and, despite its appearance in ‘The doors of perception’ (Aldous Huxley, 1954), it does not appear to be as prevalent in the public consciousness as LSD and psilocybin, potentially because of its lesser potency (Wolbach et al., 1962). However, recent naturalistic and online survey studies indicated that users report mental health benefits, such as reported improvements in depression and anxiety, related to its use (Agin-Liebes et al., 2021; Uthaug et al., 2021). In Basel, Switzerland, an ongoing clinical trial is directly comparing the effects of mescaline, psilocybin and LSD in healthy subjects (NCT04227756).

### DMT and ayahuasca

DMT has become increasingly widely used in Western society in recent years (Winstock et al., 2013), both as the vapourised and inhaled form and as a psychoactive component of the hallucinogenic brew, ayahuasca. Although similar to LSD and psilocybin in its molecular composition and affinity for the 5-HT2A receptor (Rickli et al., 2016), DMT also possesses other unique characteristics (Garcia-Romeu et al., 2016), having been identified in human bodily fluids and in rats’ pineal gland (Barker et al., 1981). Indolethylamine N-methyltransferase (INMT), the enzyme synthesising DMT from tryptamine, is widely found in the human body, although its physiological role is still unclear (Garcia-Romeu et al., 2016).

Used for millennia by Amazonian indigenous groups for medicinal and religious purposes, ayahuasca is a psychoactive plant tea, typically obtained from *Banisteriopsis caapi* and *Psychotria viridis* (Schultes and Hofmann, 1979). *B. caapi* contains beta-carboline alkaloids with monoamine oxidase inhibitor (MAOI) action, whereas *P. viridis* contains DMT. In addition to the traditional contexts, by now there is a large amount of anecdotal evidence of Western individuals having healed their depression, anxiety, addiction, PTSD and other trauma, and more through ayahuasca (e.g. Grob et al., 1996), highlighting the importance of further studying these promising effects scientifically. Palhano-Fontes et al.’s (2019) recent RCT supports the safety and therapeutic properties of ayahuasca, dosed within an appropriate setting, to help treat depression.

## Adverse effects of psychedelics

Psychedelics have come a long way since the first wave of experimentation and research. However, their potential range of psychological and psychiatric, as well as physiological risks remains to be fully understood. Table 1 provides an overview of key potential adverse effects of psychedelics, focusing on those which still loom large in public perceptions. We explore the evidence base for these adverse effects to elucidate which of these are merely based on anecdotes versus those that stand up to close scrutiny with current scientific methods.

**Table 1.** Potential adverse effects of psychedelics.

Psychological and psychiatric	Physiological
Hallucinogen use disorder	Toxicity, overdose risk
Abuse liability and dependence	Neurotoxicity
Hallucinogen-induced disorders	Hypertension, cardiovascular disease
Harms to self/others	Emergency medical treatment
Challenging experiences	
Hallucinogen persistent perception disorder	

## Psychological and psychiatric risks

### Hallucinogen use disorder

In the 1960s, the perception that psychedelics cause a special type of dependence, defined as ‘period use amongst arty types’, contributed to their strict international scheduling. Psychedelics were considered to have high abuse potential simply because there were frequent reports of their use (Isbell and Chrusciel, 1970).

The *Diagnostic and Statistical Manual of Mental Disorders*, (5th edition; *DSM-V*) acknowledges psychedelic use only as it pertains to hallucinogen persisting perception disorder (HPPD, outlined below), hallucinogen use disorders (HUDs) and hallucinogen-induced disorders (including psychotic and depressive disorders). HUDs fall under three broad categories: other HUD, hallucinogen dependence and hallucinogen abuse. HUD is described in *DSM-V* as a problematic pattern of hallucinogen use (other than phenylcyclohexyl piperidine; PCP) leading to clinically significant impairment or distress. Diagnostic criteria are summarised in Panel 1. (In *Diagnostic and Statistical Manual of Mental Disorders*, (4th edition; *DSM-IV*), this category was called HUDs.)

### Panel 1: Other HUD – *DSM*-*V* diagnostic criteria

1. The hallucinogen is often taken in larger amounts or over a longer period than was intended.
2. There is a persistent desire or unsuccessful efforts to cut down or control hallucinogen use.
3. A great deal of time is spent in activities necessary to obtain the hallucinogen, use the hallucinogen or recover from its effects.
4. Craving, or a strong desire or urge to use the hallucinogen.
5. Recurrent hallucinogen use resulting in a failure to fulfil major role obligations at work, school or home (e.g. repeated absences from work or poor performance related to hallucinogen use; hallucinogen-related absences, suspensions or expulsions from school; neglect of children or household).
6. Continued hallucinogen use despite having persistent or recurrent social or interpersonal problems caused or exacerbated by the effects of the hallucinogen (e.g. arguments with a spouse about consequences of intoxication; physical fights).
7. Important social, occupational or recreational activities are given up or reduced because of hallucinogen use.
8. Recurrent hallucinogen use in situations in which it is physically hazardous (e.g. driving an automobile or operating a machine when impaired by the hallucinogen).
9. Hallucinogen use is continued despite knowledge of having a persistent or recurrent physical or psychological problem that is likely to have been caused or exacerbated by the hallucinogen.
10. Tolerance, as defined by either of the following:

 (a) A need for markedly increased amounts of the hallucinogen to achieve intoxication or desired effect.
 (b) A markedly diminished effect with continued use of the same amount of the hallucinogen.

### Dependence

Hallucinogen dependence is a separate category to HUD, based on generic substance use dependence criteria, several of which do not apply to hallucinogens. Withdrawal symptoms and signs are not established for hallucinogens, and so this criterion is not included. In hallucinogen abuse, hallucinogens are used but much less often than in hallucinogen dependence. Diagnostic criteria include a pattern of pathological use, the impairment of social or occupational functioning due to use, and duration of disturbance of at least 1 month.

Psychedelic use does not conform to the profile of clinical features representing other types of dependencies, for example, opioids (Morgenstern et al., 1994). Very few hallucinogen users experience an inability to cut down or control use, a key indicator of dependence. HUD is relatively uncommon, with a low risk of development following exposure to hallucinogens (Shalit et al., 2019). The vast majority of hallucinogen users do not transition to hallucinogen dependence (Stone et al., 2006).

In Anthony et al.’s (1994) classic study on problematic drug use, based on representative data from the US National Comorbidity Survey, psychedelics had the lowest rate of abuse from all drugs analysed of users who qualified for a dependence diagnosis (4.9%). The Substance Abuse and Mental Health Services Administration (SAMHSA, 2017) also ranked psychedelic use at the bottom in terms of their dependence risk, although by their estimation up to 9% of psychedelic users may develop dependence (this higher percentage may be explained by the inclusion of MDMA and PCP). According to other studies using *DSM-IV* criteria, a far lower proportion of users develop hallucinogen dependence. For example, Kendler et al. (1999) provide a 0.2% estimate of hallucinogen dependence among hallucinogen-using female twins.

Today, research has repeatedly shown that psychedelics do not cause dependence or compulsive use (Halberstadt, 2015; Johnson et al., 2018; Morgenstern et al., 1994; Nichols, 2016). The effects of psychedelics are not universally euphoric (and can be dysphoric), tolerance develops quickly, cannot be overcome by dose escalation and there is no known withdrawal syndrome (Rucker et al., 2018), indicating a low risk of dependence in line with current *DSM-V* diagnostic criteria.

Tolerance – the decreased response with repeated administration of a drug – has been reported to develop rapidly to the euphoric and psychedelic effects of hallucinogens but not to the autonomic effects, such as pupillary dilation, hyperreflexia, increased blood pressure (BP), increased body temperature, piloerection and tachycardia. Cross-tolerance exists between LSD and other hallucinogens (e.g. psilocybin and mescaline). The fast build-up of tolerance and lack of withdrawal symptoms has been repeatedly shown in the literature (e.g. Krebs and Johansen, 2013; Liechti, 2017; Nichols, 2004), except for ayahuasca, which leads to minimal tolerance (Dos Santos et al., 2012).

### Abuse liability

Johnson et al. (2018) reviewed the abuse potential of medical psilocybin according to the eight factors of the controlled substances act, highlighting its limited reinforcing effects. However, although low in comparison to other scheduled substances, psilocybin does have some limited potential for abuse. Using the Addiction Research Centre Inventory (ARCI), Johnson et al. (2018) found a major difference in the abuse potential associated with psychedelics, as compared with other substances that carry a high risk of compulsive pattern of repetitive use and abuse, as LSD and psilocybin were repeatedly found to have a substantially lower potential for use and abuse (Johnson et al., 2018). Especially, the responses to the LSD scale contained a cluster of reported negative, unpleasant responses to LSD, such as ‘I feel anxious and upset’, that are associated with a lower propensity to frequently and repeatedly self-administer (Griffiths et al., 2008).

In comparison with other psychoactive drugs, psychedelics score consistently low in their abuse potential (Fábregas et al., 2010). Psilocybin has been evaluated, together with LSD in various preclinical models of dependence and abuse potential, yielding qualitatively similar results, with no physical dependence or withdrawal (Martin, 1973). Early studies showed that drugs commonly accepted as having hallucinogenic properties are not self-administered by laboratory animals (the gold standard test for dependence potential) supporting their low dependence in humans (see detailed analysis by Griffiths et al., 1979). Griffiths et al. (1979) show that the psychedelics mescaline, 2,5-dimethoxy-4-methylamphetamine hydrochloride (DOM), 2,5-dimethoxy-4-ethylamphetamine hydrochloride (DOET) and 4-methoxyamphetamine hydrochloride (PMA) did not maintain self-administration in laboratory animals, whereas other hallucinogens, for example, PCP (phencyclidine) did. This finding was further confirmed in a detailed review by Carroll (1990) who found that PCP is a highly effective reinforcer in animals, whereas LSD and other hallucinogens are not. Griffiths et al. (1979) concluded that the reinforcing effects of PCP are most likely unrelated to its hallucinogenic properties, and that the lack of self-administration in animals agrees with the finding that people use psychedelics at a very low level and that most discontinue use spontaneously. In support of this early work, a recent study in three baboons showed that, under daily schedules, they self-administered very low amounts of LSD, considerably less than cocaine. This did increase in two of these non-human primates under intermittent schedules, although still at a much lower level than cocaine (Goodwin, 2016).

Looking at psilocybin, Gable (1993) concluded that it carries a lower dependence risk than caffeine, and being among the lowest risks of death of all major substance abuse categories. In relation to ayahuasca, Gable (2006) found no evidence of abuse potential and compared its safety margin to codeine, mescaline or methadone. Rather, long-term psychological benefits have been documented when ayahuasca is used in a well-established social context. Yet, while Gable (2006) suggests that the dependence potential of oral DMT and the risk of sustained psychological disturbance are minimal, Winstock et al. (2013) argue that the very desirable effect profile of smoked DMT indicates a high abuse liability which may be offset by a low urge to use more. Similarly, administration of LSD results in high acute drug liking ratings but no craving (Holze et al., 2021; Schmid et al., 2015).

Investigating the potential abuse risk in regular ayahuasca users, Fábregas et al. (2010) used the addiction severity index (ASI) to assess addiction severity between rural and urban users in Brazil, concluding that the ritual use of ayahuasca, as assessed with the ASI in currently active users, was not associated with the negative psychosocial effects caused by other drugs of abuse.

In summary, although there have been isolated case reports of abuse (e.g. Modak et al., 2019), the characterisation of psychedelics as addictive is based on misinformation and misunderstanding. In fact, today these compounds are more often discussed in terms of their anti-addictive properties (e.g. Bogenschutz et al., 2015; Johnson et al., 2017).

### Harms to self and other

As emotional experiences can be intensified when under the influence of psychedelics, set and setting are crucial. Set and setting – the expectations and personal experiences of the users as well as the external environment – are established elements of psychedelic research and recognised as having a major impact on users’ experience (Aday et al., 2021; Johnson et al., 2008). In unprepared individuals and/or in unsafe settings, effects of psychedelics may have the potential to escalate into dangerous behaviour (Johnson et al., 2008). Although very rare, there are reports of individuals jumping from buildings and ending their lives (e.g. Honyiglo et al., 2019; Keeler and Reifler, 1967). While these occurrences are uncommon compared with other psychoactive drugs – especially alcohol – they are widely reported in the media which contributes considerably to public perceptions of their risks.

In contrast, scientific research consistently assesses psychedelics as much less harmful to the user as well as to society compared to alcohol and almost all other controlled substances. In their seminal comparative drug harms studies, using Multi-Criteria Decision Analysis (MCDA), Nutt et al. (2010) ranked LSD among the drugs with the lowest harms, both for the individual and to society and ‘magic mushrooms’ received the lowest overall harm score (Nutt et al., 2010). These findings have been replicated in the Netherlands (Van Amsterdam and Van den Brink, 2010, Europe (Van Amsterdam et al., 2015) and Australia (Bonomo et al., 2019). Carhart-Harris and Nutt’s (2013) survey of both substance users and other experts, again placed LSD and psilocybin in the lowest harm categories, and Morgan et al.’s (2010) survey of drug users further confirmed these findings.

In Carbonaro et al.’s (2016) online survey about challenging experiences after consuming ‘mushrooms’, 11% of users reported putting themselves or others at risk of physical harm. This was often related to greater (estimated) dosage, difficulty of the experience and lack of physical comfort and social support – all of which can be controlled under clinical conditions.

### A challenging experience

An adverse reaction to psychedelics can include a ‘bad trip’ (in lay language) or a ‘challenging experience’ (in therapeutic language). Although there is no exact definition of such an experience, most involve feelings of fear, anxiety, dysphoria and/or paranoia, making it essential that the experience is prepared for, supervised and followed by extensive integration. These experiences are usually short-lived, that is, lasting the time of the experience, and are often found to be cathartic.

Recent qualitative research sheds light on some of these experiences, moving them away from their negative perception, highlighting their potentially positive outcomes (Gashi et al., 2021). In Carbonaro et al.’s (2016) survey, 39% of the respondents rated their ‘worst bad trip’ as one of the five most challenging experiences of their lifetime – yet the degree of difficulty was positively associated with enduring increases in well-being. Griffiths et al. (2006) found that in a controlled study of healthy volunteers, high doses of psilocybin created extreme fear in 30% of participants, yet 80% of these participants also reported subsequent improvements in well-being. Similarly, in healthy volunteers administered high doses of LSD of 100 and 200 μg in a controlled setting, fear (with ratings > 50% on a visual analogue scale) is reported in approximately 20% and 30% of participants, respectively. Notably, more than 90% of the participants report good drug effects (> 50%) in the same session (Holze et al., 2021; Schmid et al., 2015). Recent clinical research also suggests that unpleasant reactions (such as anxiety, paranoia and confusion during the psychedelic experience) tend to be transient and do not diminish the therapeutic benefits of psychedelics in reducing depressive symptoms (Carhart-Harris et al., 2016).

Further research is required because the exact knowledge of what causes a challenging experience and who is susceptible to these experiences remains scarce. A recent systematic review found that individuals high in the traits of absorption, openness and acceptance as well as a state of surrender were more likely to have positive experiences with psychedelics, whereas individuals low in those domains or in a preoccupied/apprehensive state were more likely to experience acute adverse effects (Aday et al., 2021). Importantly, there were no sex differences, and increased age and experience with the drugs was related to slightly less intense effects. Similarly, effects of LSD were not influenced by sex or body weight in a pooled study of 81 healthy subjects. However, genetic polymorphisms of the CYP2D6 enzyme – responsible for breaking down many commonly used medicines – significantly influenced the pharmacokinetics and in part also the subjective effects of LSD (Holze et al., 2021).

Setting is likely a key influence of the progress of a psychedelic experience, as is the dose used, with a higher dose more likely to lead to these experiences (Johnson et al., 2014). Understanding the specific circumstances and individuals in which psychedelics may lead to challenging experiences will have important implications for future clinical research and harm reduction strategies.

### Serious mental health effects, including psychosis and suicide

Fears of psychedelics leading to psychosis date back to the move to ban LSD, emphasising cases of ‘acid casualties’, which had a powerful impact on society’s representations of psychedelics, although these instances are rare, especially in clinical use (see Table 2).

**Table 2.** Pooled papers of serious adverse effects.

Reference	Drug and subjects	Relevant findings
Cohen (1960)	25,000 LSD drug sessions. 5000 subjects and 25,000 drug sessions.	Suicide attempts by 1.2/1000 LSD patients; completed suicides by 0.4/1000 patients with a mental health disorder. Mescaline patients experienced agitation and very low BP and pulse, but no serious adverse events.
Chandler and Hartman (1960)	700 LSD-25 sessions.	One case of prolonged psychosis
Ling and Buckman (1963)	350 outpatients over 4 years, using LSD.	One attempted suicide
Malleson (1971)	4000 LSD patients, 50,000 sessions.	Three suicides in patients with a mental health disorder. One death of a neurotic asthmatic male, 12 h after third LSD session (dose 100 μg) One sudden death after seventh LSD session (dose 200 μg) but no organic abnormalities.

LSD: d-lysergic acid diethylamide.

For example, Cohen (1960) found one single case of a psychotic reaction lasting more than 48 h, out of 1200 experimental, non-patient research participants administered LSD or mescaline. This individual was the identical twin of a patient with schizophrenia, who would have been excluded from the research under current conditions. McGlothlin and Arnold (1971) reported one case (out of 247 participants) in which an LSD-related psychotic episode lasted more than 48 h. Although very rare, it is important to be attentive to these negative experiences and to develop enhanced safety protocols accordingly.

Earlier studies sometimes neglected the importance of set and setting, contributing to the risk of adverse effects occurring (e.g. Malitz et al., 1960; Rinkel et al., 1960) and did not include the stringent control conditions or groups that are standard in today’s clinical psychopharmacology research (Johnson et al., 2008). Adverse patient outcomes were often the result of unethical scientific methods, including restraining patients during the experience and administering high doses of LSD to unprepared, restrained patients (e.g. Smart et al., 1966). With present safety protocols for psychedelic research, such occurrences are significantly less likely, although individual cases of serious adverse effects can and do occur.

In non-clinical settings, there have been rare cases of psychedelics triggering psychotic episodes (e.g. Dos Santos et al., 2017; Tapia et al., 2021). In Carbonaro et al.’s (2016) survey, for three users (i.e. 0.15% of participants), the experience was reported to be associated with the onset of prolonged and distressing psychotic symptoms. However, this study specifically sought out individuals who had negative experiences with the drug.

This risk is greatly reduced with psychiatric screening. Those with a predisposition towards psychotic illnesses (i.e. personal or family history of schizophrenia or bipolar disorder) are generally excluded from clinical treatment with psychedelics (Johnson et al., 2008). With such screening, no psychotic episodes have been documented in modern clinical trials to the best of our knowledge.

In their systematic review, Zeifmann et al. (2021) examined the relationship between classic psychedelics and suicidality. Results suggest that psychedelic therapy can reduce suicidality in certain clinical psychiatric populations, and that classic psychedelic use may buffer against, and be associated with reductions in suicidality. However, in unsafe and unmonitored settings, psychedelic use can, on rare occasions, also lead to fatal consequences, including suicide, but Zeifmann et al. (2021) stress the highly limited nature of their source literature, with the majority of the included studies being case studies or small case reports using retrospective data.

Two large-scale population studies, each comprising over 130,000 US adults, and using data from the National Survey on Drug Use and Mental Health (NSDUH), found no evidence of an association between psychedelic use and mental health problems (Johansen and Krebs, 2015; Krebs and Johansen, 2013). Johansen and Krebs (2015) found that psychedelic users were no more likely to have experienced psychological distress, suicidal thoughts or behaviour, depression, anxiety or to have received mental health treatment in the past year than those who had not taken psychedelics. In contrast, people who had used psychedelics were less likely to have required mental health treatment in the past year than those who had not. Hendricks et al. (2015) report similar findings. The evidence for serious adverse events remains low and recent RCTs using psychedelics in various non-psychotic psychiatric disorders are showing good evidence for both safety and efficacy (Carhart-Harris et al., 2021; Palhano-Fontes et al., 2019).

### HPPD

A common perception linked to psychedelics is that they induce ‘flashbacks’ of the drug experience long after its acute effects have subsided. Although transient drug-free visual experiences resembling the effects of hallucinogens have been documented in psychedelic users (e.g. 40–60% of users; Baggott et al., 2011; Carhart-Harris and Nutt, 2010), they are not hallucinogen-specific, as they can also be caused by other psychoactive substances, for example, alcohol or benzodiazepines (Holland and Passie, 2011), and can occur in healthy populations (Halpern et al., 2016). In most cases, these side effects are mild and diminish in duration, intensity and frequency with time (Strassman, 1984).

If these symptoms are prolonged and distressing, the syndrome is known as HPPD. The *DSM-V* (American Psychiatric Association (APA), 2013) reports a prevalence rate for HPPD as 4.2% in hallucinogen users (Baggott et al., 2011) based on a single online questionnaire. Other studies have documented much lower prevalence rates of the disorder, some as low as 1/50,000 (Grinspoon and Bakalar, 1979). Furthermore, if approximately 1/25 users experience HPPD as suggested by Baggott et al. (2011), then it would be a near statistical certainty that some participants in the current era of psychedelic research, which has collectively included thousands of participants in trials since 2000 (Carhart-Harris et al., 2021; Ross et al., 2016), would have experienced HPPD by now; however, this has not been the case.

However, the emergence of large online fora dedicated to the discussion of HPPD on websites, such as Reddit (e.g. https://www.reddit.com/r/HPPD/, which has > 7000 members), suggests that cases can be identified at the population level, even if the prevalence is too low to be captured in clinical trials that typically use small sample sizes. While the large-scale data collection of online fora is helpful to gain insights into wider populations, samples are self-selected and likely to be biased, limiting the conclusions that can be drawn.

The incidence of HPPD appears to be much lower in the clinical context, perhaps as a result of efficient screening and preparation (Cohen, 1960; Johnson et al., 2008). Although Halpern and Pope (2003) suggest that there may be no identifiable risk factors for HPPD, a subsequent study of 19 individuals who developed HPPD found that all recalled anxiety and/or panic reactions during the triggering episode (Halpern et al., 2016). Thus, HPPD symptoms could potentially be conceived as a form of trauma response, similar to PTSD, or a form of health anxiety evoked by residual symptoms of the original experience.

## Physiological risks

### Neurotoxicity

A prevailing public belief about psychedelics is that they are neurotoxic (Presti and Beck, 2001). Much of this discourse has roots in the first era of psychedelic research in the mid-20th century, where several studies purported that users exhibited neurological or cognitive deficits (Acord, 1972; Acord and Barker, 1973); and others suggested that psychedelics (LSD in particular) damaged chromosomes (Cohen et al., 1967). Intriguingly and in contrast to this idea, Germann (2020) proposes the ‘psilocybin telomere hypothesis’ which states that psilocybin has a positive effect on leucocyte telomere length, which could reduce genetic ageing. In many cases, these earlier studies were refuted and retracted (e.g. Cohen et al., 1967; Dishotsky et al., 1971; Egozcue et al., 1968). Unfortunately, this did not generate the same media attention as the original work (Strassman, 1984), meaning that earlier studies played a major role in shaping media representations of psychedelics, ultimately shaping public opinion.

Most researchers now consider classic psychedelics to be non-toxic, that is, they do not damage mammalian organ systems, and as physiologically safe, even in very high doses (Gable, 2004; Halpern et al., 2005; Halpern and Pope, 1999; Malcolm and Thomas, 2021; Nichols, 2004). No long-term neurocognitive deficits have been reported by participants in the contemporary era of research (please see Aday et al., 2020b for a recent review). In a cross-sectional study, Doering-Silveira et al. (2005) compared adolescent ayahuasca users with matched non-user controls using a battery of neuropsychological tests and found no neurological deficits in users. Other studies comparing ayahuasca users with matched controls have documented *increased* working memory and executive functioning in users (Bouso et al., 2012), supporting the idea that psychedelics have neuroplastic and neurogenic properties (Catlow et al., 2013; Jefsen et al., 2020; Ly et al., 2018). DMT induces the proliferation of neural stem cells, migration of neuroblasts and generation of new neurones in the hippocampus of mice leading to improvements in working and recognition memory (Morales-García et al., 2017, 2020). These effects may explain why their therapeutic effects are so long-lasting (Carhart-Harris et al., 2016; Magaraggia et al., 2021) although further human mechanistic studies are required.

### Overdose toxicity

Psychedelics are physiologically safe in humans when ingested at standard doses (Dos Santos et al., 2012; Gasser et al., 2014; Nichols, 2004; Nichols and Grob, 2018). Overdose deaths have occurred due to ingestion of very large doses, that is, more than 23 times the previously recommended LSD human dose (Lim et al., 2012; Nichols and Grob, 2018; Van Amsterdam et al., 2011) or by mixing psychedelics with other drugs and/or alcohol (Gable, 2004; Van Amsterdam et al., 2011). For a summary of overdose and toxicity events reported in the literature, please see Table 3.

**Table 3.** Overdose and toxicity effects.

Reference	Design	Subjects	Drug	Relevant findings
Nichols and Grob (2018)	Case Studies	Five cases; 14-, 28- and 30-year-old males and a 20-year-old female. One gender and age unknown.	LSD	One death by an LSD overdose equivalent to 320 mg, two by asphyxiation and one from LSD toxicity – HR increased to 164 bpm, BP to 122/64 and temperature to 98.2°F, but toxicological report indicated 1 ng/ML of LSD which equates to only 100 μg of LSD. One female with an increase in temperature of 105°F – death by multi-organ failure, hyperthermia, and possible LSD intoxication.
Van Amsterdam et al. (2011)	Case Studies	10 cases; eight males and two females (aged between 18 and 33 years).	Magic Mushrooms (inc. *Psilocybe baeocystis* and Hawaiian psilocybin)	Three deaths from jumping of high buildings, three by overdose, one from cold temperature after large consumption of psilocybe subcubensis, one death presumed heroin overdose alongside consumption of 10 psilocybin mushrooms and one death after consumption of approx. 50, mostly raw magic mushrooms.
Cohen (1960)	Survey	Questionnaires from 44 of 62 clinicians who treated healthy 23 to 44-year-old subjects or patients with LSD or mescaline on more than 25,000 occasions	LSD doses ranged from 25 to 1500 μg and mescaline ranged from 200 to 1200 mg	Suicide attempts by 1.2/1000 LSD patients; completed suicides by 0.4/1000 patients with a mental health disorder. Mescaline patients experienced agitation and very low BP and pulse, but no serious adverse events.
Malleson (1971)	Survey	4300 patients with psychosis received approx. 49,000 sessions and 170 experimental subjects received 450 sessions – all aged between 21 and 41 years.	Doses ranged from 25 to 1500 μg; most received 100–200 μg	Three suicides in patients with a mental health disorder. One death of a neurotic asthmatic male, 12 h after third LSD session (dose 100 μg) One sudden death after seventh LSD session (dose 200 μg) but no organic abnormalities.

LSD: d-lysergic acid diethylamide.

In rats, psilocybin has been reported to have an LD50 of 280 mg/kg (Cerletti, 1958, as cited in Passie et al., 2002). This is over 700 times the high dose of 25 mg used in clinical studies, for an average body weight of 70 kg. LSD has also been shown to be safe with very low physiological toxicity (Nichols, 2016). However, there have been cases of death by overdose of psychedelics with the majority from LSD (Fysh et al., 1985; Nichols and Grob, 2018) and psilocybin (Lim et al., 2012; Van Amsterdam et al., 2011) – probably because these are the most widely used. Supplemental Appendix 1 provides a summary of these and other case reports. Older reports of administration of LSD or mescaline in a clinically supervised setting have found adverse effects or death due to the person’s underlying health conditions, such as, manic-depressive illness, acute asthma and depersonalisation syndrome (e.g. Cohen, 1960; Malleson, 1971). Yet, equally there are reports of ingesting large quantities of LSD with successful recovery and without long-lasting effects (Nichols and Grob, 2018).

Long-term ritual consumption of ayahuasca is not toxic or harmful to adults (Dos Santos, 2013) or in adolescents (Doering-Silveira et al., 2005). Doering-Silveira et al. (2005) also found no foetal deaths or abnormalities in mothers who used ayahuasca during pregnancy. However, large, well-conducted longitudinal trials in pregnant women would be needed to confirm these findings. One feature of ayahuasca, enhancing its safety profile, is the side effect of nausea and vomiting, especially at high doses (Dos Santos et al., 2012; Riba and Barbanoj, 2005; Van Amsterdam et al., 2011) which may prevent continued drug administration and overdose.

### Cardiovascular pathology in human studies

Psychedelics can induce short-lived and non-clinically significant sympathomimetic effects, including on heart rate, BP, pupil size and body temperature, as shown in Table 4.

**Table 4.** Hypertension and cardiovascular effects.

Reference	Subjects	Drug and design	Relevant findings
Schmid et al. (2015)	16 healthy participants (eight males/eight females), aged between 25 and 51 years Half were hallucinogen naïve; half had limited experience. No history of psychiatric disorders.	Single LSD (200 μg) or placebo. A double-blind, randomised placebo crossover trial.	Participants monitored for 24 h. BP peaked at 1.75 h to 140/83, HR peaked at approx. 1 h 60–80 (bpm) and body temperature peaked to 37.8°C at 3 h. No significant increase in adverse effects from 24 h post-dosage.
Holze et al. (2020)	28 healthy participants aged between 25 and 45 years (14 females/14 males).	LSD (0.1 mg), MDMA (125 mg), D-amphetamine (40 mg) and placebo. Double-blind, placebo-controlled, cross-over design.	At approx. 1 h post-LSD, BP increased to 135/81 mm Hg, HR to 86 bpm and body temperature to 37.3°C. Approx. 1.5 h after MDMA, BP increased to 140/85 mm Hg and HR to 80 bpm. Body temperature increased to 37.4°C at 4 h. D-amphetamine increased BP to 153/94 mm Hg at 0.5–1 h, HR rose to 72 bpm at 6 h and body temperature to 37.2°C at 3 h.
Gasser et al. (2014)	11 old patients aged 39–64 years (seven males/four females) with anxiety associated with life-threatening diseases (majority had cancer; one had Parkinson’s disease, one celiac disease and one Bechterew’s disease). One patient with previous LSD experience.	LSD 200 μg (experimental dose) and 20 μg (active placebo). A double-blind, active placebo-controlled, RCT.	LSD did not significantly alter BP or HR. At 0 h, post-LSD mean HR (bpm) was 74.8 and 73.3 at 8 h. Mean BP was 130.3/80.0 at 0 h and 126.3/77.5 at 8 h.
Holze et al. (2021)	16 healthy participants aged between 25 and 52 years (eight males/eight females).	LSD (0.025, 0.05, 0.1 and 0.2 mg) and placebo. Double-blind, placebo-controlled, cross-over design.	LSD significantly increased heart rate at 0.1 and 0.2 mg, reaching 81 bpm at highest dose. BP increased at doses of 0.05 mg or higher, approx. 134/83 mm Hg at dose 0.2 mg.
Dolder et al. (2016)	24 healthy participants aged between 25 and 60 years (12 males/12 females).	LSD 0.1 mg and placebo	Compared with placebo, LSD moderately increased BP, heart rate and body temperature.
Carbonaro et al. (2018)	20 healthy participants aged between 22 and 43 years (11 females/9 males). All had history of psychedelic drug use.	Double-blind design. Single, acute doses of dextromethorphan (DXM) 400 mg/kg, psilocybin 10, 20, and 30 mg/70 kg and placebo.	BP increased to 138/80 mm Hg after 10 mg/70 kg psilocybin, 142/85 mm Hg after 20 mg/70 kg and 140/87 mm Hg after 30 mg/70 kg. BP peaked to 144/85 mm Hg after DXM. 30 mg/kg psilocybin increased HR at 94 bpm and DXM at 103 bpm Max effects occurred between 2 and 4 h and decreased after 6 h.
Dos Santos et al. (2012)	Nine healthy male volunteers with experience in psychedelic drug use.	Double-blind cross-over placebo-controlled clinical trial. Dose 0.75 mg DMT/kg body weight and a placebo.	For approx. 15–30 min, SBP rose to 141 mm Hg in three volunteers after one dose and 146 mm Hg for two volunteers. In two volunteers, it rose after two doses of 147 and 142 mm Hg. DBP values did not reach above 90 mm Hg and HR rose to 105 bpm in one volunteer after one dose.
Strassman et al. (1996)	13 healthy 22- to 45-year-old experienced hallucinogen users (nine males/four females)	Randomised double-blind study design. Volunteers received four infusions of 0.3 mg/kg DMT fumarate IV or saline placebo at 30 min intervals on two separate days.	At D1 HR 99/bpm and at D4 77/bpm. Mean arterial BP peaked to 116 mm Hg at first DMT infusion and 112 mm Hg at fourth DMT infusion. Both resumed to normal within approx. 30 min.
Riba and Barbanoj (2005)	6 male volunteers in pilot study and 18 (15 males/3 females) in final double-blind trial. All healthy experienced psychedelic users.	Single-blind design for pilot, used doses of 0.5, 0.75 and 1.0 mg DMT/kg body weight. In final, double-blind randomised design doses used were 0.6 and 0.85 mg DMT/kg.	With results combined, 6 out of 24 volunteers experienced hypertension (SBP values over 140 mm Hg for two volunteers and DPB values over 90 mm Hg for four volunteers) after doses 1 mg/kg DMT in pilot and 0.85 mg/kg DMT in final study. One volunteer experienced tachycardia with HR increasing to 100 bpm.
Moreno et al. (2006)	Nine 26- to 62-year-old subjects (seven males/two females) with *DSM-IV* – defined OCD.	Four single doses of psilocybin; 100, 200 and 300 μg/kg. A very small dose of 25 μg/kg was administered randomly at any time after the first low dose. A double-blind, active placebo-controlled, randomised clinical trial.	One subject experienced transient hypertension 132/90, 135/90, 142/105, 142/95 and 116/78 mm Hg at 0, 1, 4, 8 and 24 h, respectively after 200 μg/kg of psilocybin.
Durante et al. (2020)	614 participants aged between 18 and 55 years (321 males/293 females). 50 self-reported a psychiatric disorder (commonly depression and anxiety). 31 reported using psychiatric medication – analysis considered only SSRIs and SSNIs	Cross-sectional study. Self-reported questionnaire of ayahuasca use in religious context.	Tachycardia occurred occasionally in 200 participants with higher frequency in patients with a psychiatric diagnosis (at least occasionally: 62% vs 40.07%, *p* = 0.003). No significant difference in adverse effects between participants who used antidepressants and those who did not.
Olbrich et al. (2021)	24 healthy volunteers aged 20–34 years (19 males/5 females)	Double-blind placebo-controlled RCT. Volunteers were divided into three groups. Placebo + placebo (179 mg mannitol and aerosil 1 mg) or Placebo + LSD (100 μg) or LSD + Ketanserin (40 mg).	LSD intervention increased HR, Ketanserin decreased HR, compared to the placebo condition. HR increased to approx. 60 bpm for placebo + placebo condition. For Placebo + LSD, HR increased to 70 bpm and for Ketanserin + LSD, HR increased to approx. 56 bpm.
Hasler et al. (2004)	Eight healthy 22- to 44-year-old volunteers (four males/four females).	Within-subject study design. Each volunteer received placebo and four different doses of psilocybin in random order on five experimental days at least 2 weeks apart: very low dose 45 μg/kg; low dose 115 μg/kg; medium dose 215 μg/kg and high dose 315 μg/kg.	BP peaked at 60 min to approx. 150/93 mm Hg after exposure to high dose. DBP significantly elevated up to 90 min (*p* < 0.05) SBP increased significantly at 60 min (*p* < 0.001) following HD Psilocybin. No change in body temperature or cardiac from baseline up to 24 h after drug administration.

LSD: d-lysergic acid diethylamide; MDMA: 3,4-methylenedioxymethamphetamine; DXM: Dextromethorphan; DMT: dimethyltryptamine.

Most studies examined involved healthy subjects, some included patients with anxiety, or OCD, and in one large study of participants in ayahuasca ceremonies, a small number were taking antidepressant medication. Schmid et al. (2015) found that LSD induced a small but significant increase in BP, heart rate and body temperature in a sample of 16 healthy volunteers with normal values restored at 24 h post-dosing. Other studies reported similar results for LSD (Dolder et al., 2016; Gasser et al., 2014; Holze et al., 2020, 2021), psilocybin (Carbonaro et al., 2018), ayahuasca (Dos Santos et al., 2012) and DMT (Strassman et al., 1996). Combined results from Riba and Barbanoj’s (2005) double-blind pilot study and clinical trial with ayahuasca found that 6 out of the 24 volunteers in their study met the diagnostic criteria for hypertension during drug administration and one volunteer had tachycardia. However, no medical assistance was required, and participants’ symptoms subsided.

However, 200 out of the 641 participants taking part in Durante et al.’s (2020) study experienced tachycardia, and frequency of occurrence was higher in patients with a psychiatric diagnosis than those without. However, it is unclear if this was due to direct effects of ayahuasca or a result of participants’ underlying psychiatric disorder and/or medication. No difference in adverse effects was found between participants who used antidepressants and those who did not (31 participants reported using antidepressant medication). However, the combination of MAOIs, such as that found in ayahuasca, with SSRIs has the potential to lead to serotonin syndrome (Gillman, 2010), highlighting the importance of educating ayahuasca drinkers of this potential risk.

Overall, adverse effects are observed at higher doses (Cohen, 1960), risks are substantially increased when mixed with other substances (Gable, 2004; Van Amsterdam et al., 2011) and mortality is more common in patients with physical or mental health disorders, such as acute asthma and manic-depressive illness, than in healthy subjects (Cohen, 1960; Malleson, 1971). These risks are greater when drugs are used in unsupervised settings. With adequate inclusion and exclusion criteria and clinical supervision, adverse physiological reactions are minimal (Malleson, 1971; Muttoni et al., 2019).

Even in non-supervised setting, adverse effects remain rare. Looking at the self-reported incidence of emergency medical treatment (EMT) sought for LSD and ‘magic mushrooms’, EMT is consistently low, and less than 1% of users report seeking help (Global Drug Survey (GDS), 2019). In comparison to other recreational drugs, psychedelics rank as the lowest in the United States, with 1.9 emergency department visits per 100,000 in 2011 (Substance Abuse and Mental Health Services Administration (SAMHSA), 2017). In relation to hospital admissions, SAMHSA (2017) shows that the rate of ‘hallucinogens’ as the primary substance is at 0.1% of hospital admissions.

A recent Freedom of Information request to the Office for National Statistics (ONS, 2021) confirms the remarkably low overdose rate of LSD and psilocybin. Based on deaths registered in England and Wales (between 1993 and 2020), there were eight deaths where LSD was specified on the death certificate and two deaths where psilocybin was mentioned, with one death certificate reporting the presence of both substances. As mentioned above, mixing psychedelics with other drugs and/or alcohol can have detrimental effects, including death (Van Amsterdam et al., 2011). The dose (Gable, 2004), route of administration and likelihood of any underlying health condition/s (Malleson, 1971) also determine potential adverse effects, such as multi-organ failure, hyperthermia and intoxication leading to other risky behaviours (Nichols and Grob, 2018; Van Amsterdam et al., 2011).

### The quality of available evidence

When evaluating the potential risks of psychedelic medicines as scientifically and objectively as possible, it is important to acknowledge that some of the evidence presented above (particularly studies conducted pre-prohibition) is not of the highest standard as described by Rucker et al. (2016) in their recent review. But today’s scientific-technological approaches have advanced considerably since the early research. For an example of current techniques applied to enable our understanding of how psychedelics produce their effects, please see Singleton et al. (2021). Most earlier shortcomings are being addressed in recent trials, that is, in randomised placebo double-blind studies (Carhart-Harris et al., 2021; Mitchell et al., 2021).

We have included evidence from both eras in an attempt to incorporate large evidence based on the safety of psychedelics. The approach of psychedelic-assisted psychotherapy (PAP) to psychiatric drug development is unique, a paradigm shift in fact. Therefore, this may not need to comply with the standard protocols required to enable a new chemical entity (NCE) to reach patients with a fully evaluated safety profile. Shahid et al. (2020) provide an extensive description of this process from drug target selection to testing in animal models, Phase I to Phase IV clinical studies to post-marketing surveillance and risk management. PAP drug development currently involves plant medicines that have been used safely by indigenous populations for thousands of years, by western populations over successive generations and currently in clinical trials for many psychiatric disorders in controlled situations. Such molecules do not require the same development steps as NCEs, as considerable information regarding their safety and efficacy already exists. However, as pharma becomes involved in PAP drug discovery to develop new psychedelic molecules with improved drug delivery systems, absorption, distribution, metabolism and excretion (ADME) profiles and reduced potential for toxicity in vulnerable populations, the processes described by Shahid et al. (2020) may become a requirement.

Furthermore, post-marketing surveillance and risk management, that is, pharmacovigilance, will likely gain in importance. As psychedelics are currently being given in non-clinical settings to patients and healthy volunteers (e.g. in retreats) and remain unlicensed as a medicine, pharmacovigilance currently rests with online for a qualitative investigations and organizations, such as the Psychedelic Experience (https://www.psychedelicexperience.net/). Once licenced formally, however, pharmacovigilance can proceed as for any new medicine to further assess patient safety (Shahid et al., 2020).

### Summary

The physiological safety of psychedelics is by now relatively well established, and they have been described as ‘one of the safest known classes of CNS drugs’ (Nichols, 2016: 275). Psychological and psychiatric effects are less predictable and although rare, serious reactions can occur. Johnson et al. (2008) conclude that psychedelic use may involve unique psychological risks, the most common being participants having a challenging experience, while prolonged psychoses and HPPD are far less likely.

Table 5 summarises differences between clinical versus non-clinical uses and users. While categories are blurred and use and users might be overlapping between categories, and there are many similarities between medical and non-clinical uses (such as that neither are likely to be using daily or for users to become dependent), notable differences can be discerned in users’ set and setting, as well as the pre- and aftercare experienced both areas where above adverse effects are potentially exacerbated. Psychedelics can induce a vulnerable state, not just during but also after use (Andersen et al., 2021).

**Table 5.** Clinical versus non-clinical use/users.

Clinical	Non-clinical
Safe and reliable dosing	Unknown dosing
Quality assurance of substance	Unknown quality of substance
Within controlled healthcare setting	Unknown setting
Full screening of patients	Screening limited or non-existent
Individual patients	Usually group setting
Medical support available	Medical support usually unavailable
Pre- and aftercare by trained psychologists, psychotherapists and so on.	Pre- and aftercare limited or non-existent. Fear of criminalisation may lead to unwillingness to seek medical help
No repeated dosing	More frequent dosing common
Aim to heal underlying disorder	Aim to heal/well-being/ spirituality/intoxicate/fun
Non-daily use	Non-daily use

Within the clinical environment, set and setting, as well as the overall care experienced, can be largely controlled (Rucker et al., 2018). Rapport between patient and therapist is vital as the patient is undergoing a potentially life-changing experience (for many, with a substance they have no previous experience with) especially as co-creating truly informed consent between providers and patient can be challenging (Andersen et al., 2021). Training and experience of the therapists (both during the dosing sessions and for the all-important integration sessions) is also essential (Tai et al., 2021).

The importance of preparation, supervision and integration work, as well as general emotional support – be that in a clinical or traditional ceremonial context – cannot be overestimated (e.g. Kettner et al., 2021) and a range of approaches to ensure psychedelic harm reduction and integration of challenging experiences are being developed (e.g. Gorman et al., 2021). Patient safety and well-being must always come first, together with a full appreciation of responsibility to develop outstanding standards of clinical training, quality assurance and peer review (Andersen et al., 2021).

## Conclusion

Some of the perceived harms of psychedelics – for example, that they lead to addiction and are neurotoxic – are largely refuted by research of the past decades. Other risks, such as the risks of psychotic episodes or overdose, are rare and only reported in individual cases, but these risks still need to be minimised by careful patient selection and preparation. The past decade of research and clinical experience has increasingly demonstrated how psychedelics can be used safely under medical supervision, and safe use guidelines are progressively well defined (e.g. Griffiths et al., 2006).

Regulatory and legal hurdles of getting psychedelic medicines proven as mainstream medicines are still substantial, so overcoming historic misperceptions is vital. The past decade has seen an increasing focus on research on the therapeutic applications of psychedelics – a direct benefit for the public, which is positively represented in current media (Aday et al., 2019). A recent YouGov study (2017) indicates that public perceptions in the United States becoming more positive, with the majority (63%) being open to medical treatment with psychedelics if faced with a pertinent medical condition, and a UK YouGov survey (2021) corroborates these results.

These changes in public interest are in line with the recent regulatory changes in the United States and Canada. Collectively, these changes in public perception and regulation suggest that the stigma surrounding psychedelics may be beginning to dissipate, and that society is moving away from previous negative narratives to a more scientific, evidence-based approach to risks and benefits of psychedelics as medicine.

## Supplemental Material

sj-docx-1-jop-10.1177_02698811211069100 – Supplemental material for Adverse effects of psychedelics: From anecdotes and misinformation to systematic science Click here for additional data file. Supplemental material, sj-docx-1-jop-10.1177_02698811211069100 for Adverse effects of psychedelics: From anecdotes and misinformation to systematic science by Anne K Schlag, Jacob Aday, Iram Salam, Jo C Neill and David J Nutt in Journal of Psychopharmacology
