# Supplementary material for: Adverse effects of psychedelics: From anecdotes and misinformation to systematic science
Source: J Psychopharmacol. 2022 Feb 2;36(3):258–72. doi: 10.1177/02698811211069100 (PMC8905125; doi:10.1177/02698811211069100)
Supplement: sj-docx-1-jop-10.1177_02698811211069100 – Supplemental material for Adverse effects of psychedelics: From anecdotes and misinformation to systematic science [file sj-docx-1-jop-10.1177_02698811211069100.docx]

**Appendix 1 - Overdose and Toxicology related Case studies**

| Reference | Design | Subjects | Drug | Relevant findings |
| --- | --- | --- | --- | --- |
| Fysh et al., (1985) | Case Study | 25-year-old male | LSD (unknown amount) | Patient died 16 hrs after being admitted into hospital. Unclear how much and exactly when the LSD was ingested. Analysis of post-mortem serum showed 14.4 ng/ml. |
| Lim, Wasywich and Ruygrok (2012) | Case Study | 24-year-old heart transplant female recipient. | Magic mushrooms (Unknown quantity) | 9-years post-transplant Patient collapsed 2-3 hrs after consuming an unknown quantity of magic mushrooms. Plasma toxicology levels were 30ug/L and tetrahydrocannabinol level of 4 ug/L. Cause of death determined as psilocin toxicity. |
| Klock et al., (1974) | Case Studies | 4 women and 4 men. 19 to 39 years old | LSD tartrate ranged from 2.1 to 26 nanograms per ml and 1,000 to 7,000 mg per 100 ml, respectively. | Transient hypertension was present in three patients with highest values reaching 230/130. Heart rate varied from 110 to 200 bpm.  Blood concentrations of LSD for four patients were 26 ng/mL and gastric concentrations 7.0 mg/100 ml. All patients recovered within 12 hours. |
| Haden and Woods (2020) | Case Studies | (AV) 15-year-old female with bipolar disorder. (NM) 26-year-old pregnant female. (CB) 46-year-old female with Lyme disease. | Accidental overdoses of 1000 μg, 500 μg and 55 mg of LSD. | All patients survived.  AV experienced significant reduction in bipolar disorder symptoms for almost 20 years.  NM who was pregnant in her first trimester did not experience any negative effects during pregnancy. Her child did not experience teratogenic or other negative developmental effects - 18-years-old at the time of report.  CB experienced positive effects on pain levels and morphine withdrawal. |
| Giancola et al., (2020) | Case Study | 30-year-old man with bipolar disorder type I | injected psilocybin mushrooms intravenously | BP 75/47 and HR 100 bpm. Survived but on long term regimen of medicines for multiple system organ failures. |
| Nef et al., (2009) | Case Study | 17-year-old male | Psilocybe semilanceata (dose unknown) | BP: 120/60 mmHg and HR 93 bpm.  Acute coronary syndrome which normalised after 6 days |

**Appendix 2 Search strategy Details**

| **Databases and restrictions** | |
| --- | --- |
| Databases | PubMed, Google Scholar, UoM library and PsychInfo |
| Duplicates | Checked for duplicates via built-in functions and by screening titles and abstracts. |
| Reference list | Relevant in text references and screening of reference list |
| inclusion criteria’s | From 1960 – 2020  English language  Peer-reviewed  Journal articles  non-English were excluded. |
| Dates | Oct 2020 -Nov 2020 and general more specific searches afterwards in databases and through reference lists |

| **Search Strategy Iterations** | |
| --- | --- |
| Psychedelic | Hallucinogen / classic hallucinogens  Classical psychedelics / psychedelics  Ayahuasca / DMT /Dimethyltryptamine / Harmine  Psilocybin / Psilocin  LSD / Lysergic acid diethylamide |
| physiological | Cardiovascular (effects)  tachycardia  Blood pressure  hypertension |
| Toxicological effects | Toxic* (effects)  Overdose |
| Other | Adverse effects  Clinical trial  Double-blind randomised trial  Human  Survey  Literature review  Systematic review |
